# A Conjugate between Lqh-8/6, a Natural Peptide Analogue of Chlorotoxin, and Doxorubicin Efficiently Induces Glioma Cell Death

**DOI:** 10.3390/biomedicines10102605

**Published:** 2022-10-17

**Authors:** Lucie Dardevet, Feten Najlaoui, Sonia Aroui, Mayeul Collot, Céline Tisseyre, Michael W. Pennington, Jean-Maurice Mallet, Michel De Waard

**Affiliations:** 1 L’institut du Thorax, Nantes Université, CNRS, INSERM, 44000 Nantes, France; 2 LabEx “Ion Channels, Science & Therapeutics”, 06560 Valbonne, France; 3 Laboratoire des Venins et Biomolécules Thérapeutiques LR11IPT08, Institut Pasteur de Tunis, Tunis 1002, Tunisia; 4 Laboratory of Biochemistry, Molecular Mechanisms and Diseases Research Unit, UR12ES08, Faculty of Medicine, University of Monastir, Monastir 09023, Tunisia; 5 Laboratoire de Bioimagerie et Pathologies, UMR CNRS 7021, 74 route du rhin, CS 60024, 67401 Illkirch, France; 6 Université Grenoble Alpes, 621 Avenue Centrale, 38400 Saint-Martin d’Hères, France; 7 Ambiopharm Inc., 1024 Dittman Crt, North Augusta, SC 29842, USA; 8 Laboratoire des Biomolécules, Ecole Normale Supérieure, PSL University, Sorbonne University, CNRS UMR 7203, 75005 Paris, France; 9 Smartox Biotechnology, 6 rue des Platanes, 38120 Saint-Egrève, France

**Keywords:** chlorotoxin, Lqh-8/6, glioma cells, apoptosis, doxorubicin, conjugation, click chemistry

## Abstract

Natural peptides isolated from animal venoms generally target cell surface receptors with high affinity and selectivity. On many occasions, some of these receptors are over-expressed in cancer cells. Herein, we identified Lqh-8/6 as a natural peptide analog of chlorotoxin, a proven and useful compound for the diagnosis and treatment of glioma. Lqh-8/6 and two other natural analogues were chemically synthesized for the first time and evaluated for their ability to label, detect and prevent glioma growth in vitro. We demonstrate that a biotinylated version of Lqh-8/6 allows both the labeling of glioma cell lines and the detection of glioma in brain sections of glioma allograft Fisher rats. Lqh-8/6 has intrinsic anti-invasive properties but is non-toxic to glioma cells. To confer anti-tumor properties to Lqh-8/6, we chemically coupled doxorubicin to the glioma-targeting peptide using click chemistry. To this end, we successfully chemically synthesized Lqh-8/6-azide and doxorubicin-alkyne without impairing the toxic nature of doxorubicin. The toxin-drug conjugate efficiently promotes the apoptosis of glioma cells in vitro. This example contributes to the concept that animal venom peptides constitute exquisite warheads for delivering toxic chemical conjugates, a parallel to the popular concept of antibody-drug conjugates for the treatment of cancer.

## 1. Introduction

Gliomas are primary brain tumors derived from glial cells [1]. Fifty five percent of these gliomas are represented by glioblastomas (GBM), which are the most aggressive form of glioma [2]. The median survival time of patients affected by GBM is 12–15 months [3]. Just in the USA, 12,120 new cases of patients affected by GBM are diagnosed each year (estimate for 2016) [2,4]. GBMs are derived from astrocytes [5,6]. The core of a GBM tumor mass is composed of highly undifferentiated tumor cells that end up in necrosis, whereas the boundaries of the tumor mass are more heterogeneous in terms of dedifferentiation level. Neovascularization is more pronounced at the boundaries of the tumor mass and significant levels of healthy tissue infiltration are evidenced [7]. There is no preventive diagnosis possible, and generally, patients consult a neurologist upon the manifestation of a neurological disorder. The nature of the deficit is of course correlated with the exact localization of the tumor mass in a given brain region. Final diagnosis is established by magnetic resonance imaging (MRI) and biopsy coupled to histopathology characterization if possible [8]. New neuroimaging modalities are continuously developed to assess the response to treatment as well [9].

Patient treatment first involves the exeresis of the tumor mass by a neurosurgeon, which represents a complex surgical intervention for two reasons: (i) the localization and size of the tumor does not always allow the safe removal of the tumor mass; (ii) the boundaries of the tumor mass are difficult to define with precision for the surgeon because of the infiltrating nature of GBM into healthy surrounding tissue [10]. The patient medical management also involves a combination of chemotherapy associated to irradiation [10,11]. Temolozomide is considered as the gold standard for chemotherapy in treating GBM [12,13]. Thanks to these medical efforts, the percentage of patients that pass the five-year survival time rises from 1.9 to 9.8% [14]. In comparison, with the progress of oncology treatment, patients affected by prostate and breast cancers now have a five-year survival time above 50%. There is thus considerable progress to be made for the treatment of GBM. Reasons for the failure of successful GBM management involve (i) incomplete resection during surgery for the reasons mentioned above; (ii) limited passage of the temolozomide across the blood–brain barrier, a general problem for chemotherapy; and (iii) the weak radiosensitivity of GBM cells. 

There is thus a clear need for the development of innovative therapies and management of GBM patients. There are several research and clinical initiatives around the world that attempt to fill the gap in the management of GBM compared to other cancer types. One initiative, called “Orphanet” (www.orpha.net), that classifies GBM as an orphan disease due to its low occurrence in the general population lists the worldwide ongoing projects. As of August 2022, there are 127 clinical assays ongoing, of which 17 are international and 110 are national. Most of the efforts are concentrated on immunotherapy, which is a heavy trend in the management of several cancer types [15,16]. The most advanced projects include anti-GBM vaccination and immune checkpoint inhibitors [15]. While industrial efforts generally focus on immunotherapy, there are also several projects developed in academia that concentrate their efforts on tumor vectorization for the targeted delivery of anti-tumor agents. Two trends have appeared: (i) the use of GBM targeting peptides (GTP); (ii) the use of Tumor-Penetrating Peptides (TPP). The later are meant to not only penetrate tumor cells, but also to possess some preferential GBM-targeting properties. Alternatively, TPP may also carry a function that annihilates a GBM pathway that is selectively present only in GBM cells, so that the cell targeting function is not essential [17,18,19,20].

One particular GTP has encountered frank success: chlorotoxin [21]. Chlorotoxin is a 36 amino acid peptide from the venom of the Israeli scorpion *Leiurus quinquestriatus quinquestriatus* and is folded according to a αβββ motif. These secondary structures are connected internally by four disulfide bridges [22,23]. It was first used as a pharmacological tool, like many other natural peptides before, for the study and characterization of chloride channels (ClC-3, small-conductance Cl^−^ channels) [24,25,26,27,28]. Soon after, with the understanding that there is a specific chloride current present in glioma cells (GCC, Glioma-specific Chloride Channels), blocked by chlorotoxin, it was discovered that chlorotoxin was capable of targeting glioma, GBM, melanoma, small cell lung carcinoma, neuroblastoma, and medulloblastoma cancer cells (all of neuroectodermal origin) [29,30]. It should be mentioned, however, that the membrane receptor of chlorotoxin still needs formal confirmation because it was also found to bind onto Matrix Metalloprotease MMP-2 and annexin-A2 [31,32]. The compound was patented by TransMolecular Inc. in 2002 for its GBM-targeting properties. Since then, many derivatives have been produced with the aim of covering various applications in oncology. Examples include: (i) TM-601, ^131^I-chlorotoxin up to phase III clinical trials (phase I/II completes in 2003 and authorization for phase III in 2006); (ii) the infrared-sensitive Cy5.5-chlorotoxin to help surgeons delineate the boundaries of the GBM tumors [33]; and (iii) a variety of functionalized nanoparticles [34,35,36,37,38]. Chlorotoxin was also used as a toxin-drug conjugate, which mirrors the use of antibody-drug conjugates [39]. It was conjugated to a chelator of platinum for the efficient delivery of platinum into GBM cells and the induction of apoptosis. Since the acquisition of the chlorotoxin patent license or patent itself by Morphotek Inc., a subsidiary of EISA Corp., little information is now published on the commercial and clinical fate of chlorotoxin.

As chlorotoxin has become so popular for the study of GBM treatment and diagnosis, researchers have neglected to look for the existence chlorotoxin-related toxins that would present the same diagnostic and therapeutic potential than chlorotoxin. This would, however, be advantageous to circumvent the intellectual property rights and further our understanding of the structural determinants that make chlorotoxin so efficient. A closer look at the taxonomy of scorpion species indicates the existence of two subspecies of *Leiurus quinquestriatus* and presupposes the existence of several chlorotoxin-like toxins [40]. A sequence homology search using chlorotoxin as entry reveals the existence of fifteen known scorpion toxins that present at least a 61% sequence identity with chlorotoxin (Table 1). All these toxins possess between 34 and 38 amino acids. They are all connected according to a C_1_-C_4_, C_2_-C_6_, C_3_-C_7_ and C_5_-C_8_ disulfide bridging pattern. The amino acid regions that present the greatest diversities are the C-terminus and the domain between C_5_ and C_7_. BmKCT, a peptide from the venom of the scorpion *Buthus martensi*, with a 76% sequence identity with chlorotoxin, was recently shown to inhibit cell division and the migration of GBM cells, as well as to possess anti-angiogenic properties [41,42], clearly indicating that the search for functional homologs of chlorotoxin is not in vain.

**Figure 1:**
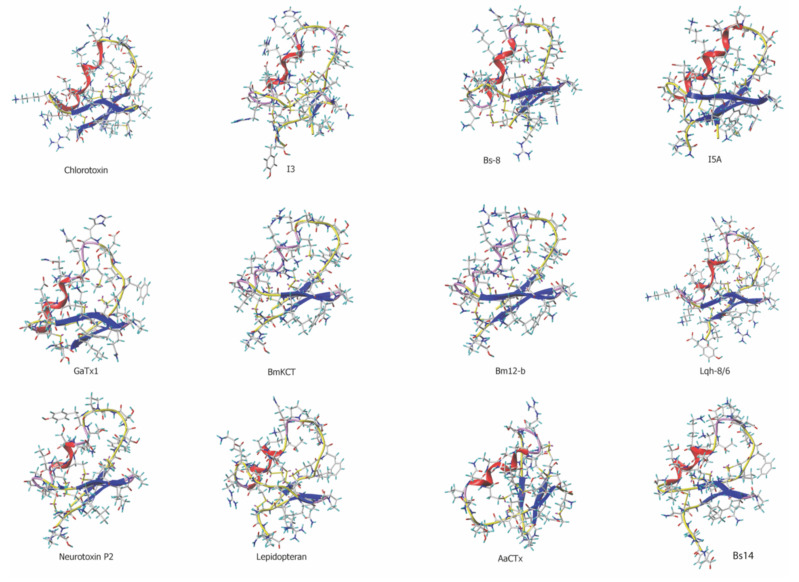
Three-dimensional structure of chlorotoxin and chlorotoxin-like peptides. The 3D structure of chlorotoxin and I5A were obtained from their pdb files: 1CHL (chlorotoxin) and 1SIS (I5A). All other structures are models built by homology with chlorotoxin using @TOME V2 and modeler for Lqh-8/6, Bs-8, Bs-14, I3, GaTx1, BmKCT, Bm12-b, neurotoxin P2, AaCTx and lepidopteran. All toxins present an anti-parallel β-sheet structure and most an alpha helix (with the exception of BmKCT and Bm12-b).

By using the program @TOME V2 [43], we built homology models for these toxins (Figure 1). A clear compact structural homology is evident from these toxin representations with the presence of α-helix combined with an antiparallel three-stranded β-sheet (see also [44]). The secondary structures were well conserved, as illustrated by a superimposition of peptide backbones (Supplementary Figure S1). One of the most conserved analogues is Lqh-8/6, a toxin isolated from the venom of a subspecies of *Leiurus quinquestriatus* (*hebraeus* instead of *quinquestriatus*). It has a 72% sequence identity with chlorotoxin. This percentage is lower than the sequence identities observed between chlorotoxin and several other analogues: BmKCT (76%), I_3_ and I_4_ (82% both), Bs-8 (80%) and I_5_ and I_5A_ (79% both). However, one distinguishing feature of Lqh-8/6 compared to the other analogues is that many substitutions are conservative (Lys^14^, Lys^25^, and Ile^34^ instead of Arg^14^, Arg^25^, and Leu^34^, respectively). In addition, there is a sequence extension at the C-terminus that lowers the sequence homology compared to other toxins. Finally, the comparison of the modeled structures illustrates that Lqh-8/6 most closely resembles the structure of chlorotoxin.

Herein, we chemically synthesized Lqh-8/6 for the first time by solid-phase peptide synthesis by producing two variants, a biotinylated one and an analogue compatible with click chemistry for the grafting of doxorubicin, an anti-tumor agent. We demonstrate the ability of the peptide to selectively bind to GBM rat F98 cells both in vitro and in situ from the brain slices of rats containing F98 allografts. We also show that Lqh-8/6 does not present cell toxicity and or affect cell migration. However, it significantly reduces cell invasion. We chemically produced doxorubicin-alkyne for the click chemistry conjugation to azide-Lqh-8/6 with the rationale to benefit from the Lqh-8/6 targeting properties combined to the antitumor activity of doxorubicin. The resulting hybrid molecule was tested on two glioma cell lines: rat F98 and human brain U-87 glioblastoma astrocytoma that are used classically in experimental neuro-oncology. This conjugate produces significant GBM rat F98 cell death and human U-87 caspase-3 dependent cell apoptosis. In vivo, the Lqh-8/6-doxorubicin significantly delays tumor growth from F98 allografts implanted in the striatum of Fisher rats. Globally, this project promotes the concept of natural peptide-drug conjugate for the efficient targeting and treatment of cancers, which is analogous to the antibody-drug conjugate concept but without the difficulties related to size and production.

## 2. Materials and Methods

### 2.1. Chemicals

N-α-Fmoc-L-amino acid, Wang-TentaGel resin and reagents used for peptide synthesis were obtained from Iris Biotech (Markterdwitz, Germany). Analytical-grade quality solvents (acetonitrile (ACN), dimethylformamide (DMF), N-methylpyrrolidone (NMP), trifluoroacetic acid (TFA)) were from Acros Organics (Illkirch, France). For Western blotting, the antibodies used were anti-caspase-3 (H-277) and anti-β-actin (B-6) (Santa Cruz Biotechnology Inc., Heidelberg, Germany); anti-Bax (Merk Millipore, Darmstadt, Germany); anti-Bim (BD transduction Laboratories, Erembodegem, Belgique); and peroxidase-conjugated goat anti-rabbit and anti-mouse antibodies (Jackson Immunoresearch Laboratories, West Grove, PA, USA). z-VAD-fmk and z-DEVD-fmk, used to study apoptosis, were obtained from Sigma-Aldrich (Lyon, France). Doxorubicin hydrochloride was a gift from Peptides International (Louisville, Kentucky, USA). Hoechst 34580, concanavalin A-rhodamine, concanavalin A-alexa 647, and Cy5- or Cy3-streptavidin (strep-C5 or strep-Cy3) were purchased from Thermo Fisher Scientific (Waltham, MA, USA). Thiazolyl Blue Tetrazolium Bromide (MTT) was obtained from Sigma-Aldrich. LIVE/DEAD^®^ Viability/Cytotoxicity Kit for mammalian cells was used for toxicity measurements (Thermo Fisher). The CytoSelect™ 96-Well Cell Migration and Invasion Assay Combo Kit, 8 µm, was to evaluate cell migration and invasion (Cell Biolabs Inc., San Diego, CA, USA).

### 2.2. Molecular Modeling

Using the Sybyl X 1.3 software (Tripos Inc., St. Louis, MO, USA) and the Protein Data Bank (PDB) structure of chlorotoxin (code 1CHL) and @Tome2 web server [43], we generated 3D models of Lqh8/6, Bs-14, I3, GaTx1, BmKCT, Bm12-b, Neurotoxin P2, AaCTx and lepidopteran. Several steps of minimization and control of the stereochemistry were performed to obtain a model for each molecule. The parameters of the minimization were as follows: field force and charge: MMFF94, method: mathematical algorithm of Powell without initial optimization, termination point: when gradient = 0.5 kcal/(mol.Å).

### 2.3. Peptide Syntheses

Chemical syntheses of the various peptides (Lqh-8/6, Lqh-8/6-biotin (Lqh-8/6_b_), Lqh-8/6-azide, chlorotoxin, BS-14, BS-14-biotin (BS-14_b_), leptidopteran and leptidopteran-biotin (lepidopteran_b_)) were performed as previously described [45]. Biotins were added at the N-termini of peptide sequences. Briefly, peptides were chemically synthesized by the solid-phase method using an automated peptide synthesizer (CEM^©^ Liberty, Orsay, France). Peptide chains were assembled stepwise on 0.24 mEq of Fmoc-L-Arg(Pbf)-Wang-TentaGel resin using 0.24 mmol of N-α-fluorenylmethyloxycarbonyl (Fmoc) L-amino-acid derivatives. The side-chain protecting groups were: trityl for Cys and Asn; tert-butyl for Ser, Thr, Glu and Asp; Pbf for Arg; and tert-butylcarbonyl for Lys. Reagents were at the following concentrations: Fmoc-amino-acids (0.2 M Fmoc-AA-OH in DMF); activator (0.5 M 2-(1H-benzotriazole-1-yl)-1,1,3,3-tetramethyluronium hexafluorophosphate in DMF); activator base (2 M diisopropylethylamine in NMP); and deprotecting agent (5% piperazine/0.1 M 1-hydroxybenzotriazole in DMF), as advised by the PepDriver software (CEM^©^). After peptide chain assemblies, the resins were treated 4 h at room temperature with a mixture of TFA/water/triisopropylsilane (TIS)/dithiothreitol (DTT) (92.5/2.5/2.5/2.5; *v*/*v*/*v*/*v*). The peptide mixtures were then filtered, and the filtrates were precipitated by adding cold tert-butylmethyl ether. The crude peptides were pelleted by centrifugation (10,000× *g*, 15 min) and the supernatants were discarded. The peptides were purified by Reverse-phased High Pressure Liquid Chromatography (RP-HPLC) using a Vydac C18 column (218TP1010, 4 µm, 250 × 100 mm) using a 10–60% ACN linear gradient containing 0.1% TFA. Crude peptides were then oxidized/folded in 0.1 M of Tris.HCl buffer at pH 8.3 for 48 h before purification of the folded/oxidized peptides by RP-HPLC using a Vydac C18 column (218TP104, 5 µm, 250 × 46 mm), again with a 10–60% ACN linear gradient with 0.1% TFA. The correct oxidations of the synthesized peptides were checked by MALDI-TOF mass spectrometry, whereas proper disulfide pairing was determined by trypsin digestion and mass spectrometry analyses of the fragmented peptides.

### 2.4. Cell Cultures

Undifferentiated malignant glioma rat (F98) and differentiated malignant glioma rat (RG-2) cell lines (from American Type Culture Collection (ATCC)) were maintained at 37 °C in 5% CO_2_ in DMEM/F-12 nutrient medium (Invitrogen, Cergy Pontoise, France), which was supplemented with 2% (*v*/*v*) fetal bovine serum (FBS, Invitrogen) and 100 µg/mL of streptomycin and 100 units/mL of penicillin (Invitrogen). Human glioblastoma (U-87) cell line (from ATCC) was maintained at 37 °C in 5% CO_2_ in low-glucose (1 g/L) Dulbecco’s modified Eagle’s medium (DMEM) (Invitrogen) supplemented with 10% fetal bovine serum (Invitrogen), 100 µg/mL of streptomycin and 100 units/mL of penicillin (Invitrogen). All cell media were changed every 2 or 3 days. All experiments were carried out on cells with initial viability exceeding 95%. After seeding (96- and 24-well plates or 8-well Labtek slides), the cells were incubated overnight to adhere at the surface and then treated with drugs at different concentrations and time intervals.

### 2.5. Confocal Microscope Imaging of GBM Cells

For analysis of the cell entry of Cy5-labeled-peptides in living F98 cells, cell cultures were incubated with the fluorescent peptides (in DMEM/F-12 nutrient medium only) for 5 min, 30 min or 2 h, and then washed twice with phosphate-buffered saline (PBS) alone. Cell nuclei were stained with 60 µg/mL of Hoechst 34,580 for 5 min before the cell cultures were washed with PBS. The plasma membrane was stained with 50 μg/mL of concanavalin A-rhodamine for 5 min. The cells were washed once more. The live cells were then immediately analyzed by confocal laser scanning microscopy using a Zeiss LSM operating system. Hoechst 34,580 (λ_ex_ 405 nm), rhodamine (λ_ex_ 561 nm) and Cy5 (λ_ex_ 633 nm) were sequentially excited and emission fluorescence was collected. For the 5 min incubation time with fluorescent peptides, the staining of the cell nuclei and plasma membrane were performed simultaneously. For the analysis of the subcellular distribution of doxorubicin and doxorubicin-alkyne in living cells, cell cultures were incubated for 2 h with the compounds in DMEM/F-12 nutrient medium lacking FBS, and then washed twice with PBS. Cell nuclei and plasma membrane staining were performed as described in the previous paragraph, except that concanavalin A-alexa 647 was used instead of concanavalin A-rhodamine. Live cells were then immediately analyzed by confocal laser scanning microscopy with doxorubicin and doxorubicin-alkyne being excited at λ_ex_ 590 nm.

### 2.6. Cy5-Peptide Labeling of GBM F98 Cells in Culture

An amount of 50 µL of streptavidin-Cy5 (16 µM) and 39.4 µL of biotinylated peptides (81 µM) were mixed in 310 µL of DMEM/F-12 nutrient medium only. The mixtures were vortexed and incubated for 1 h at room temperature to produce the fluorescent peptides. They were used as such for cell labeling in cell cultures.

### 2.7. Synthesis of Doxorubicin-Alkyne

Triethylamine (20 µL, 144.4 µmol, 4 eq) followed by propargyl chloroformate (10 µL, 108.7 µmol, 3 eq) was added to a solution of doxorubicin hydrochloride (21 mg, 36.26 µmol) in methanol (10 mL). The solution was stirred at room temperature for 5 min. The solvents were then evaporated and the product was solubilized in dichloromethane (DCM) and washed with HCl (1 M) and a saturated solution of NaHCO_3_. The organic phase was dried over MgSO_4_, filtered and evaporated to give 22 mg of pure doxorubicin-alkyne (97% yield) as an orange solid.

### 2.8. Conjugation of Doxorubicin-Alkyne to Lqh-8/6-azide

Lqh-8/6-azide (3 mg, 0.69 µmol, 1 eq) was dissolved in 300 µL of water/DMF (1:1), and 660 µL of doxorubicin-alkyne (10 mM in methanol/DCM, 1/1, *v*/*v*) was added (10 eq). Then, 34.5 µL of CuSO_4_.5H_2_O (100 mM in water, 5 eq) and 34.5 µL of sodium ascorbate (100 mM in water, 5 eq) were added to the mixture. The solution was sonicated and bathed at 35 °C for 2 days. The solution was then purified by RP-HPLC using a Vydac C18 column (218TP104, 5 µm, 250 × 46 mm). Elution of doxorubicin-Lqh-8/6 was performed with a 5–90% acetonitrile linear gradient containing 0.1% TFA. The pure fraction was lyophilized and quantified. An amount of 3.3 mg of doxorubicin-Lqh-8/6 was purified, representing a theoretical yield of 90%. Doxorubicin-Lqh-8/6 was characterized by MALDI-TOF mass spectrometry.

### 2.9. Excitation and Emission Spectra of Doxorubicin and Doxorubicin-Alkyne

Solutions of doxorubicin (180 µM) and doxorubicin-alkyne (370 µM) were prepared in water/dimethylsulfoxide (DMSO) (1/1, *v*/*v*). The absorbance spectra were obtained using a spectrophotometer (PHERAstar FS, BMG Labtech, Champigny-sur-Marne, France). Using the wavelength of the maximal absorbance of doxorubicin and doxorubicin-alkyne, the fluorescence emissions were recorded.

### 2.10. Stereotaxic Implantation of F98 Cells in Fisher Rat Brain Striatum

Two-month-old male Fisher rats were anesthetized by inhalation of isoflurane (induction by isoflurane 4% in air, followed by anesthesia maintenance by isoflurane 2% in air). The cranium was then shaved before placing the rats in a stereotaxic frame. After disinfecting the scalp, an incision followed by resection of the subcutaneous tissue was made. Trepanning of the skull was realized at 3.5 mm to the right of the median line with a needle of 25G. The implantation of F98 cells was performed by a slow injection (0.5 µL/min) of 5 µL of solution (10^3^ F98 cells in PBS) using a Hamilton syringe connected to a pump. This injection was made 5.5 mm below the external surface of the skull. After the slow removal of the syringe, the burr hole was filled with Horsley’s wax and the scalp stitched with absorbable thread. The rats were placed in an incubator until awakening and then monitored for 2 h before returning to the care facility. The tumor-growth was monitored by RMI. The animals were euthanized when their condition was judged too painful to keep them alive. Their brains were removed and instantaneously frozen in a bath of cold isopropane, removed and stored at −80 °C before use.

### 2.11. Cy3-Peptide Labeling of F98 GBM Tumors in Brain Slices of Allograft Rats

Frozen sections (8 µm thick) were fixed in cold methanol (100%) for 8 min at −20 °C, then washed in PBS and saturated with 10% FBS (Fisher Scientific SAS, Illkirch, France) in PBS for 60 min. The sections are incubated overnight with Lqh-8/6_b_ at 4 °C. The next day, the tissues were washed 3 times with PBS, then fixed for 2 min with 4% PFA 60% acetone and 20 mM of HEPES, washed with PBS and incubated with Cy3-streptavidin (1/1000; Fischer Scientific SAS, Illkirch, France) and Hoechst 33,258 (1/1000; Sigma-Aldrich, L’Isle d’Abeau Chesnes, France) for 2 h at room temperature. The brain sections were digitized using a DMI6000 microscope (LEICA, Nanterre, France), which was equipped with a 20× dry objective and a EMCCD Quantem camera that was driven by the MetaMorph software V7.1 (Roper Scientific, Lisses, France).

### 2.12. Migration and Invasion Assays

These assays were performed according to the manufacturer’s protocol (CytoSelect™ 96-Well Cell Migration and Invasion Assay Combo Kit, 8 µm; Cell Biolabs Inc.). Boyden chambers either contained or did not contain an extracellular matrix (ECM, type IV collagen), depending on the type of measurement required: (i) invasion (with ECM) or (ii) migration (without ECM). GBM F98 cells were seeded in the upper Boyden chambers at a density of 0.5 × 10^6^ cells/mL in a culture medium without FBS. Peptides were added immediately in the upper chamber for 24 h at 37 °C. The lower chamber contained culture medium with FBS that acted as a chemoattractant. The upper chamber was separated from the lower chamber by a polycarbonate membrane with 8 μM size pores. Cells that reached the inner part of the Boyden chamber 24 h later were dissociated by a cell detachment buffer added to the lower chamber. The invasive or migratory cells, depending on the chamber type, were then lysed and quantified using CyQuant GR fluorescent Dye. The fluorescence of the lysates was read using a plate reader at λ_ex_ 480 nm/λ_em_ 520 nm (PHERAstar FS, BMG Labtech SARL, Champigny sur Marne, France).

### 2.13. MTT Assays

The cells were seeded into 96-well plates at a density of 8000 cells/well. After 24 h of culture, the cells were incubated for 72 h at 37 °C with chlorotoxin, Lqh8/6, Bs-14 or lepidopteran at various concentrations. Control wells containing cell culture medium with or without cells, both without peptide addition, were included in each experiment. Saponin (0.1%) was used as a toxic agent for comparison. The cells were then incubated with 3-(4,5-dimethylthiazol-2-yl)-2,5-diphenyltetrazolium bromide (MTT) for 30 min. The conversion of MTT into purple-colored formazan by the living cells indicates the extent of cell metabolism. The crystals were dissolved with DMSO and the optical density was measured at 540 nm using a microplate reader (PHERAstar FS) for the quantification of cell viability. All assays were run in triplicate.

### 2.14. GBM Cell Viability Assays by Flow Cytometry

The cells were seeded into 24-well plates at a density of 10,000 cells/well. After 24 h of culture, the cells were incubated for 72 h at 37 °C with chlorotoxin, Bs-14, lepidopteran, Lqh-8/6, doxorubicin, doxorubicin-alkyne or doxorubicin-Lqh-8/6 at various concentrations. Negative and positive controls consisted of untreated and 0.1% saponin-treated cells, respectively. After incubation, the cells were washed with PBS and detached with 500 μL of trypsin/EDTA (ThermoFisher, Illkirch, France). The cells were centrifuged at 200× *g* for 5 min and resuspended in 500 µL of staining mix (DMEM/F-12 nutrient medium without FBS with 31 nM of calcein and 1 µM of ethidium homodimer). Cell suspensions were incubated for 15 min at room temperature and then analyzed by flow cytometry. Flow cytometry analyses were performed using a C6 Flow cytometer (Accuri, BD Biosciences, Pont de Claix, France). The cells were gated by forward/side scattering from a total of 10,000 events. The data obtained were analyzed using the Accuri CFlow software (Accuri).

### 2.15. Apoptosis Pathway Induced by Lqh-8/6, Doxorubicin-Alkyne and Lqh-8/6-Doxorubicin in U-87 Cells

The Hoechst staining of cells—1 × 10^4^ cells were grown on coverslips into a 6-well plate and incubated with Lqh-8/6 (1 µM), doxorubicin (10 µM) or Lqh-8/6-doxorubicin (10 µM). Thereafter, the cells were fixed with 4% paraformaldehyde for 30 min at room temperature and washed twice with PBS. Chilled methanol (−20 °C) was added to each well for 10 min at room temperature followed by 2–3 washes with PBS. The cells were then stained with Hoechst 33,258 (5 mg/mL) for 10 min, washed with PBS and then examined by a Zeiss fluorescent microscope. Cells with condensed, fragmented nuclei and brighter fluorescence were considered as apoptotic cells. The percentage of apoptotic cells was calculated from the ratio of apoptotic cells to total cells counted. At least 300 cells were counted for each condition.

### 2.16. Western Immunoblotting Analyses

The cells were incubated for the indicated time and concentration of compounds and cultured in Petri dishes to about 75% confluence in serum-free medium supplemented with 0.1% BSA. The incubated cells were washed twice with cold PBS and scrapped in ice-cold lysis buffer (10 mM of Tris pH 7.5, 0.5 mM of EDTA pH 8.0, 0.5 mM of DTT, 0.5% CHAPS, 10% glycerol) supplemented with a cocktail of protease inhibitors. After 30 min on ice, cell debris was removed by centrifugation at 10,000× *g* at 4 °C for 20 min. Proteins were quantified using the DC Protein Assay (Bio-Rad, Marnes-la-Coquette, France) according to the manufacturer’s specifications. Twenty µg of protein lysate was incubated in loading buffer (60 mM of Tris-HCl, pH 6.8, 0.18 M of β-mercaptoethanol, 2% SDS, 10% glycerol, and 0.005% bromophenol blue), boiled and separated on a polyacrylamide gel by SDS-PAGE under reducing conditions. The proteins were electro-transferred overnight or 2 h at 4 °C to polyvinylidene difluoride (PVDF) membrane (Hybond-P, ThermoFisher Scientific, Illkirch, France). Membranes were blocked for at least 1 hr in Tris-buffered saline (TBS), 5% BSA and 0.5% Tween 20 and then probed with the appropriate primary antibodies (1:1000) in 5% BSA, 0.1% Tween 20 and 0.05% sodium azide in TBS for 1 h at room temperature or overnight at 4 °C. Thereafter, they were incubated with the appropriate secondary peroxidase-labeled antibody (1:20.000) for 1 hr at room temperature. Enhanced chemiluminescence (ECL, ThermoFisher Scientific, Illkirch, France) was used for protein detection. The ECL staining was quantified by densitometry with ImageJ software (National Institutes of Health, Bethesda, MD, USA).

## 3. Results

### 3.1. Lqh-8/6_b_ Efficiently Labels GBM Tumor Cells

**Figure 2:**
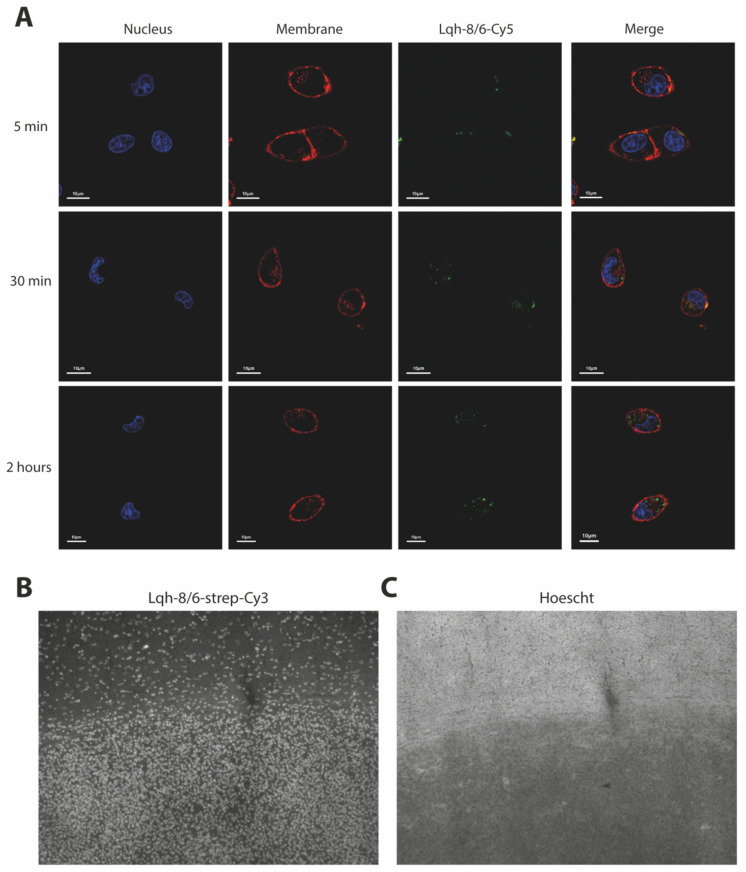
GBM F98 labeling with Lqh-8/6_b_. (**A**) Confocal microscopy images illustrating the cell surface labeling and penetration of Lqh-8/6_b_-strep-Cy5 into glioma F98 cells (green color). Incubation times were 5, 30 min and 2 h. Images were taken immediately after washout of the extracellular peptide. The plasma membrane is labeled with concanavalin-A-rhodamine (red color) and the nucleus with Hoechst 34,580 (blue color). **(B**) Brain slice of F98 tumors in allograft Fischer rat labeled with Lqh-8/6_b_-strep-Cy3. (**C**) Hoechst staining showing position of cell nuclei and density of F98 cells in brain tumor.

Based on its structural homology with chlorotoxin, we expected that Lqh-8/6 should also be able to bind onto a surface receptor of GBM cells. We first chemically synthesized this peptide in its biotinylated version (Lqh-8/6_b_), and then coupled it to strep-Cy5 in vitro to visualize peptide binding. Live F98 cells were incubated for 5 min, 30 min or 2 h with 3 µM of Lqh-8/6_b_-strep-Cy5 and the nuclei and plasma membrane of these cells stained with Hoechst 34,580 and concanavalin A-rhodamine, respectively. As shown by confocal microscopy, Lqh-8/6_b_-strep-Cy5 labeled the plasma membrane of F98 cells in vitro (Figure 2). Interestingly, toxin labeling at 5 min revealed cell surface clustering of the receptor. With longer exposure times (30 min and 2 h), internalization of the ligand/receptor complex could be witnessed. No labeling of the nucleus was evident, suggesting that the fluorescence remained restricted to the cytoplasm, and presumably, endosomal compartments. Similar experiments using BS-14_b_-strep-Cy5 and lepidopteran_b_-strep-Cy5 produced comparable F98 cell surface staining and time-dependent internalization of the toxin receptors (Supplementary Figure S2). These data indicate that Lqh-8/6 (72% sequence identity with chlorotoxin), BS-14 (61% sequence identity) and lepidopteran (63% sequence identity) all have the ability to bind to a surface receptor of F98 GBM cells, which is coherent with structural homologies between these peptides. To check the specificity of this labeling, Lqh-8/6_b_-strep-Cy3 staining was performed on 8 µm-thick coronal brain slices from Fisher rats that were previously injected with F98 cells in the striatum and in which the tumor developed for 25 days. As shown, on these coronal slices a predominant staining by Lqh-8/6_b_-strep-Cy3 was evident in the tumor zone (Figure 2B). This zone was confirmed by the evidence of a high density of nuclei (Hoechst staining) due to the lack of cell extensions such as neurites (Figure 2C). Lqh-8/6_b_-strep-Cy3 staining also illustrates the presence of Lqh-8/6-positive cells further outside the tumor zone. Their presence is probably explained by the high invasiveness of this tumor cell type, especially 25 days after tumor development. These findings illustrate the potential value of a fluorescent derivative of Lqh-8/6 for tumor painting GBM cells in situ.

### 3.2. Lqh-8/6 Lacks Intrinsic Cell Toxicity

**Figure 3:**
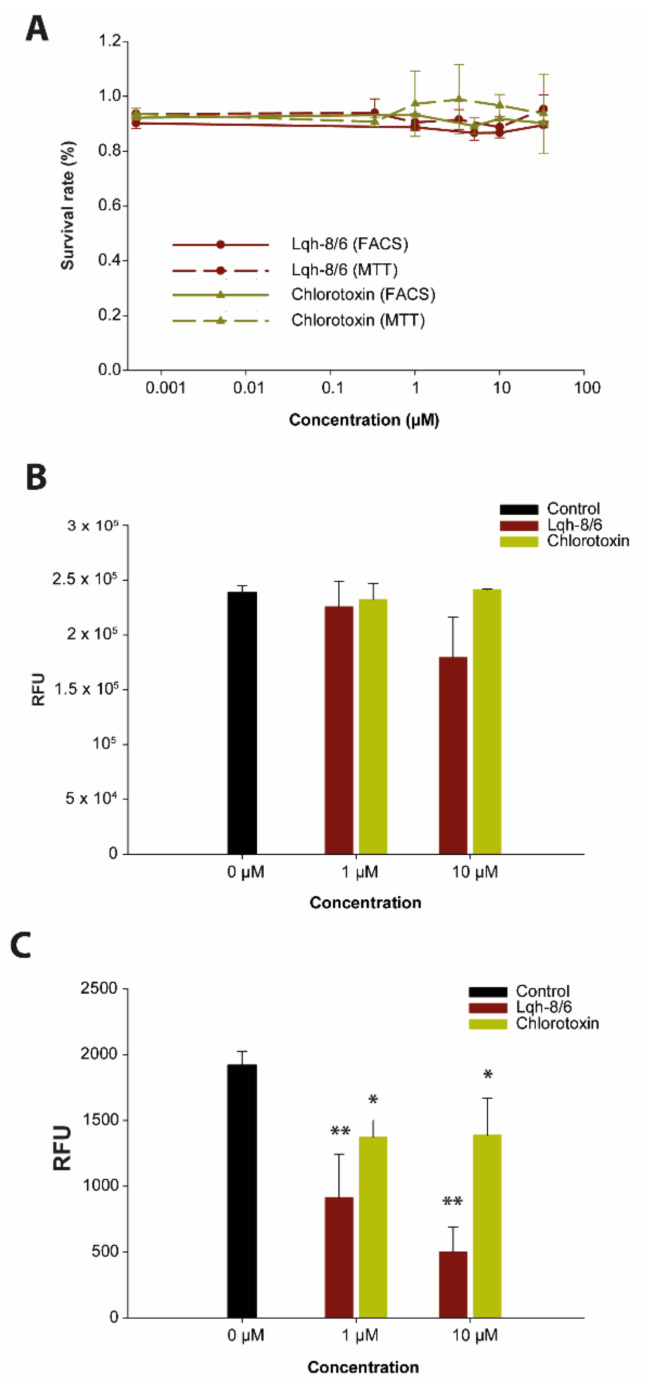
Functional effects of chlorotoxin and Lqh-8/6 onto F98 cells in culture. (**A**) Lack of cell toxicity of chlorotoxin and Lqh-8/6 as assessed by MTT assay and flow cytometry. Toxicity results from flow cytometry data (calcein for living cells and ethidium homodimer for dead cells) are represented by full lines, whereas MTT results are shown with dashed lines. (**B**) Effect of chlorotoxin and Lqh-8/6 on F98 cell migration. RFU: Relative Fluorescence Unit. (**C**) Effect of chlorotoxin and Lqh-8/6 on F98 cell invasion. * *p* ≤ 0.07; ** *p* ≤ 0.05.

Next, we assessed whether Lqh-8/6 presents any intrinsic cell toxicity. Both MTT tests and cell viability assays by flow cytometry were performed on F98 cells in vitro after 72 h of incubation with the toxins. Both tests demonstrate that Lqh-8/6, like chlorotoxin, presents no intrinsic toxicity up to 33 µM (Figure 3A). Similarly, BS-14 and lepidopteran are also non-toxic to F98 cells in vitro, demonstrating that the F98 cell surface toxin receptors are not required for cell survival and metabolism (Supplementary Figure S3A).

### 3.3. Lqh-8/6 Affects Invasion but Spares Migration

To test the effect of Lqh-8/6 on cell migration and invasion, we used Boyden chambers without and with ECM at the bottom of the chamber. As shown, Lqh-8/6 has no significant effect on F98 cell migration at low (1 µM) and high (10 µM) concentrations (Figure 3B), a result also observed for BS-14 and for lepidopteran at 1 µM (Supplementary Figure S3B). However, a significant inhibition of cell migration was observed for lepidopteran at 10 µM. Concerning invasion by F98 cells in control conditions, 0.8% of cells showed a propensity to cross the Boyden chamber after ECM degradation. Interestingly, Lqh-8/6 was capable of inhibiting F98 cell invasion at both concentrations tested (1 and 10 µM), indicating that the peptide efficiently inhibits ECM degradation by F98 cells (Figure 3C). The extent of inhibition reached 74 ± 9.8% (*n* = 3) at 10 µM of Lqh-8/6 and was superior to that mediated by chlorotoxin. Lepidopteran was even more efficient than Lqh-8/6 at 1 µM to block cell invasion, while BS-14 showed more limited efficacy at both concentrations (Figure S3C). These findings are consistent with earlier claims that chlorotoxin acts by potentially binding on MMP-2 [31], an important player for ECM degradation.

Altogether, these data demonstrate that Lqh-8/6, like chlorotoxin and other toxin analogs, have limited inherent anti-tumor efficacies (lack of cell toxicity and no major inhibition of migration). However, they have moderate to significant effects on cell invasion, combined with potent GBM-targeting properties. In the next steps, we improved the anti-tumor properties of Lqh-8/6 by grafting the peptide onto a potent cytotoxic agent with the aim of preserving the targeting properties of the peptide for selective delivery to F98 GBM cells.

### 3.4. Chemical Synthesis and Characterization of a Click Chemistry-Compatible Doxorubicin Analogue

**Figure 4:**
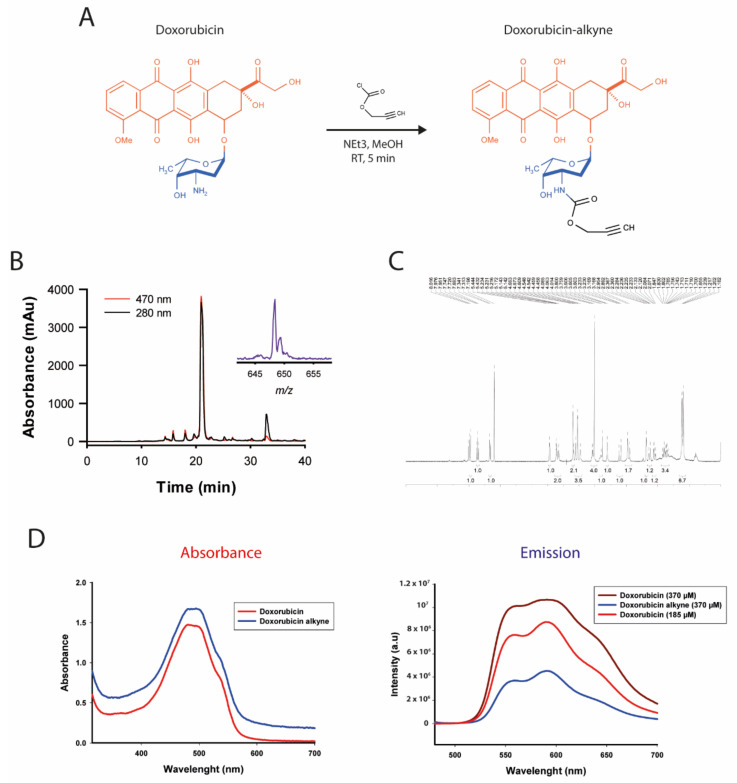
Synthesis and characterization of doxorubicin-alkyne. (**A**) Chemical synthesis of doxorubicin-alkyne. The aglycone moiety is shown in orange, while the daunosamine moiety is represented in blue. (**B**) RP-HPLC purification and mass spectra characterization of doxorubicin-alkyne. (**C**) ^1^H-NMR characterization of doxorubicin-alkyne. (**D**) Spectral properties of doxorubicin and doxorubicin-alkyne.

Doxorubicin is an anthracycline generally used for the treatment of solid cancers. It is a DNA intercalating agent and inhibitor of topoisomerase type II (both nuclear targets), but also a Reactive-Oxygen Species (ROS)-generating compound (mainly cytoplasmic target), all promoting cell apoptosis. However, it possesses significant secondary effects, and namely produces cardiomyopathy, a condition that limits its long-term use. It would therefore benefit from being grafted to a vectoring agent, thereby limiting unwanted effects on healthy tissues. Doxorubicin has two chemical functions that have been used in the past while keeping its activity intact to some extent [46,47,48,49,50]. These include: the alcohol function of the aglycone moiety or the amino group of the daunosamine moiety (Figure 4A). Earlier evidence seems to suggest that doxorubicin loses more activity if the alcohol function is functionalized rather than the amine function, probably by interfering with the intercalating properties of doxorubicin [48]. To render doxorubicin compatible for chemical grafting on Lqh-8/6, we turned towards click chemistry for its ease of use and reproducibility. We chemically produced doxorubicin-alkyne in one step by mixing doxorubicin with propargyl chloroformate in the presence of trimethylamine (Figure 4A).

The resulting product was purified by RP-HPLC and verified by MALDI-TOF MS (Figure 4B). Doxorubicin-alkyne has a molecular weight (MW) of 625.4 Da (648.3 Da with a Na^+^ adduct), compared to doxorubicin, which has a MW of 543.5 Da. The ^1^H-NMR spectrum of doxorubicin-alkyne confirms the expected structure of the synthetic product (Figure 4C). Since the N-acylation of doxorubicin has been performed in methanol—a protic solvent, which is a typical condition to avoid O-acylation—only the desired N-acylated product was obtained in a high yield (97%) with no sign of O-alkylation by ^1^H-NMR nor by mass spectrometry. This change in structure on the amine function is susceptible to alter the chemical properties of doxorubicin. We first checked whether the chemical modification had an impact on the spectral properties of doxorubicin (Figure 4D). As shown, the spectrum of absorbance of doxorubicin-alkyne was not significantly modified compared to the spectrum of absorbance of doxorubicin. In contrast, the intensity of emission fluorescence was significantly reduced by a factor of 2.5-fold at the peak of emission without a change in the wavelength-dependence of the emission. This modification of the spectral emission properties will, therefore, limit our capacity to detect doxorubicin-alkyne in cells.

**Figure 5:**
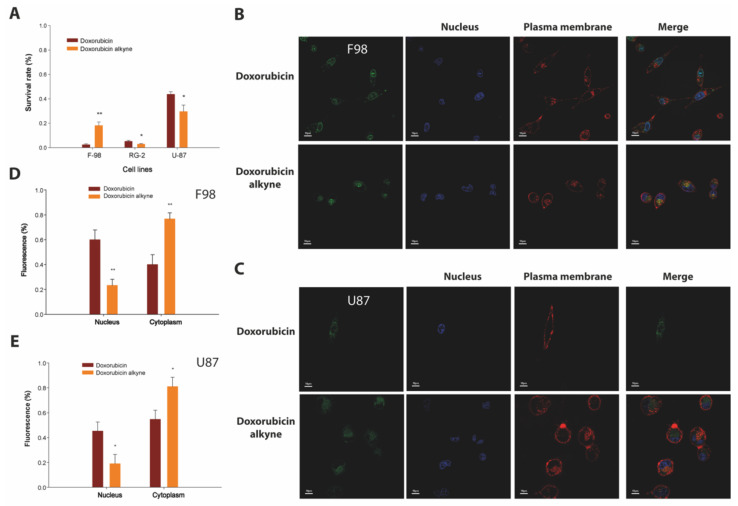
Cell toxicity on GBM cells and subcellular distribution of doxorubicin-alkyne. (**A**) Cell toxicity of 10 µM of doxorubicin and doxorubicin-alkyne incubated 72 h with various cell lines. (**B**) Cell localization of doxorubicin and doxorubicin-alkyne in F98 cells at 3 µM (2 h incubation time). Doxorubicin and doxorubicin-alkyne are shown in green. The plasma membrane is labeled with concanavalin-A-alexa fluor 647 (red color) and the nucleus with Hoechst 34,580 (blue color). (**C**) Cell localization of doxorubicin and doxorubicin-alkyne in U-87 as in (**B**). (**D**) Quantification of the cell localization of doxorubicin and doxorubicin-alkyne in F98 cells. (**E**) Quantification of cell localization of doxorubicin and doxorubicin-alkyne in U-87 cells. Scale bars in (**B**,**C**): 10 μm. *, *p* ≤ 0.05, **, *p* ≤ 0.01.

Next, we evaluated the cell-inducing toxicity and subcellular distribution properties of doxorubicin-alkyne compared to doxorubicin alone in various GBM cells. A 72 h-incubation of F98, RG-2 or U-87 GBM cells with 10 µM of doxorubicin or doxorubicin-alkyne produces significant cell toxicity, as assessed by the cell viability assay (Figure 5A).

On rat F98 cells, the alkyne modification slightly reduced the cell toxicity of doxorubicin: 18 ± 3% survival (n = six wells and N = 6 × 5000 cells, doxorubicin-alkyne) vs. 2.4 ± 0.6% survival (n = six wells and N = 6 × 5000 cells, doxorubicin). However, the opposite observation was made for rat RG-2 and human U-87 cells with doxorubicin-alkyne being slightly more potent than doxorubicin (2.9 ± 0.3% (doxorubicin-alkyne) vs. 5.1 ± 0.8% (doxorubicin) for RG-2, and 43.7 ± 2.1% (doxorubicin-alkyne) vs. 29.6 ± 5.3% (doxorubicin) for U-87 in similar conditions). Of note, human U-87 GBM cells seem intrinsically more resistant to doxorubicin treatment compared to rat cell line GBM models. These data confirm that the toxicity of doxorubicin is largely preserved despite the chemical modification. Slight differences in the cell toxicity efficiencies of doxorubicin-alkyne can be tentatively explained by alterations in the cell toxicity contribution of the different doxorubicin targets (nucleus vs. cytoplasm). Despite the limited emission efficiency of doxorubicin-alkyne, we managed to examine the relative nuclear/cytoplasm subcellular distribution of the alkyne-modified doxorubicin and compared it to the distribution of parent doxorubicin (Figure 5B–E). As shown, doxorubicin-alkyne was essentially present in the cytoplasm of F98 cells and less in the nucleus (76.7 ± 5.0%, nuclear/cytoplasm ratio = 0.31), whereas the opposite was true for doxorubicin (40 ± 7%, nuclear/cytoplasm ratio = 1.6). A similar trend for a more pronounced cytoplasmic localization of doxorubicin-alkyne was observed for U-87 cells (nuclear/cytoplasm ratio = 0.23 compared to 0.86 for unmodified doxorubicin). Reasons for these changes may include (i) a greater size of doxorubicin-alkyne, making it more difficult to cross nuclear pores, or (ii) a less pronounced DNA-intercalating efficacy. Whatever the reasons for these changes, it is concluded that doxorubicin-alkyne remains a valid anti-tumor agent that is now compatible for click chemistry coupling onto Lqh-8/6.

### 3.5. Coupling of Doxorubicin-Alkyne to Lqh-8/6-azide Produces an Efficient Anti-GBM Peptide-Drug Conjugate

**Figure 6:**
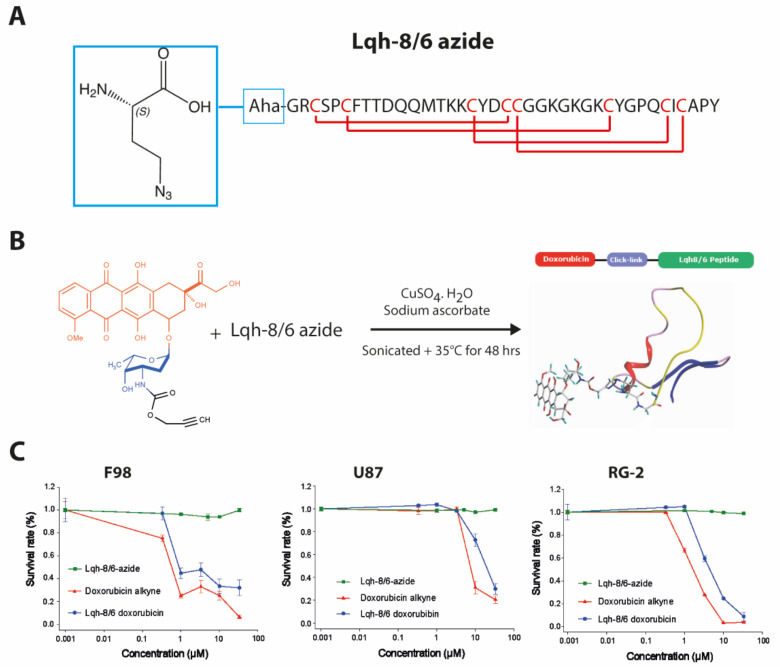
Lqh-8/6-azide primary structure, chemical coupling to doxorubicin-alkyne and GBM cell toxicity of Lqh-8/6-doxorubicin conjugate. (**A**) Amino acid sequence and disulfide bridge arrangement of Lqh-8/6-azide. Note the additional N-terminal Gly residue (boxed) and the Aha residue (larger box) aimed at spacing the peptide and doxorubicin moieties. (**B**) Huisgen cycloaddition of Lqh-8/6-azide and doxorubicin-alkyne to produce Lqh-8/6-doxorubicin. Note that the doxorubicin moiety is well spaced from Lqh-8/6 structure. (**C**) Concentration-dependence of Lqh-8/6 azide, doxorubicin azide and Lqh-8/6-doxorucin on F98 (left panel), U87 (mid panel) and RG-2 (right panel) cell survival. 72 h chronic incubation.

To follow up on the click chemistry coupling of doxorubicin-alkyne onto Lqh-8/6, we first modified the peptide sequence by grafting an azide function. We chose to add this function onto the N-terminus because, as shown earlier (Figure 2), the biotinylated version of Lqh-8/6 is efficient in labeling F98 cells despite the additional presence of biotin at the N-terminus. We therefore synthesized Lqh-8/6-azide by incorporating an additional Gly residue at the N-terminus, in order to provide a spacer, preceded by Aha (an Ala residue onto which an azide function has been added) (Figure 6A). The chemical synthesis was performed using Fmoc chemistry and the peptide was folded/oxidized similarly to Lqh-8/6 or Lqh-8/6_b_. MALDI-TOF MS confirmed that the proper mass was obtained ([M+H]^+^ = 4348.1 Da). The Huisgen 1.3 dipolar cycloaddition was used to couple Lqh-8/6-azide to a five-fold excess of doxorubicin-alkyne (Figure 6B). CuSO_4_ was used as a Cu(I) donor and sodium ascorbate as an activator. A representation of the resulting compound is shown, illustrating that doxorubicin is not hindered by the size of Lqh-8/6.

Next, the conjugated Lqh-8/6-doxorubicin peptide was evaluated for its expected propensity to affect GBM cell viability assay by FACS. Three cell lines were studied: rat F98, human U-87 and rat RG-2). For the sake of comparison with Lqh-8/6-doxorubicin, the effects of Lqh-8/6-azide and doxorubicin-alkyne alone were also evaluated in parallel. As shown, while Lqh-8/6-azide has no toxicity per se, similarly to Lqh-8/6 alone (Figure 3A), the conjugate displayed significant cell toxicity on all three cell lines (Figure 6C). Of interest, the concentration-dependence of the toxic effects of the conjugate paralleled the concentration-dependence of the toxic effects of doxorubicin-alkyne, but always showed a shift towards higher concentrations. This is expected, since doxorubicin is now attached to a peptide that also needs to bind to a plasma membrane receptor and enter GBM cells through a receptor/ligand internalization process. The IC_50_ values measured for the conjugate were 0.84 ± 0.05 µM (F98), 16.6 ± 0.05 µM (U-87) and 4.14 ± 0.03 µM (RG-2). In comparison, the values observed for doxorubicin-alkyne alone were 0.34 ± 0.02 µM (F98), 9.22 ± 0.06 µM (U-87) and 1.67 ± 0.02 µM (RG-2), indicating that the conjugation and alteration of the cell entry mechanism leads to moderate efficacy decreases in the dose-response efficacies of 2.47-, 1.8- and 2.48-fold, respectively.

### 3.6. Lqh-8/6-Doxorubicin Induces GBM Cell Death by Apoptosis

**Figure 7:**
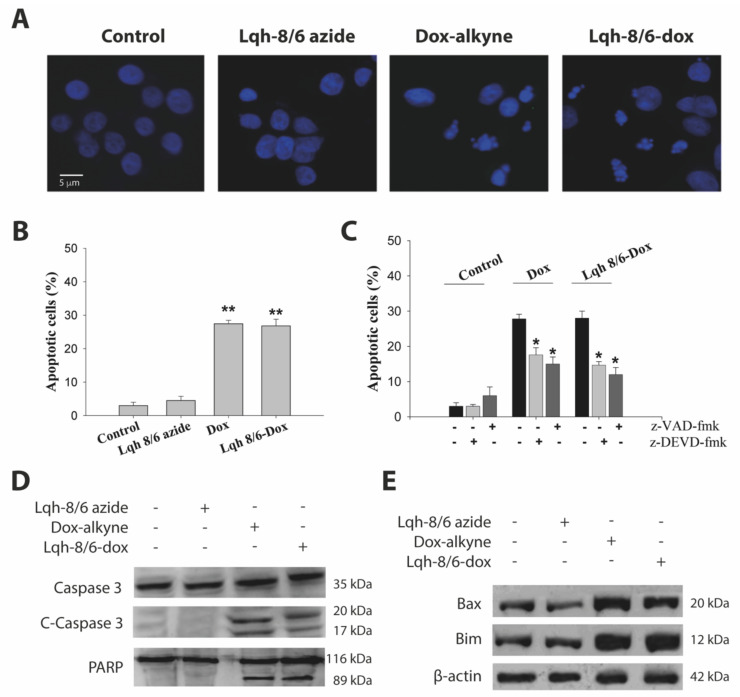
Lqh-8/6-doxorubicin induces U-87 cell apoptosis. (**A**) Hoechst-stained U-87 cells showing morphological changes induced in the cell nuclei. Apoptotic cells show condensed and fragmented nuclei. Concentration: 10 μM (Lqh-8/6-azide, doxorubicin-alkyne or Lqh-8/6-doxorubicin). (**B**) Percentage of apoptotic cells induced by the various treatments for 24 h at 10 µM. **, *p* ≤ 0.01. (**C**) Effects of a broad-spectrum caspase inhibitor, z-VAD-fmk, and a caspase-3/-7-specific inhibitor, z-DEVD-fmk, on the percentage of apoptotic cells for the different treatments. *, *p* ≤ 0.05. (**D**) Caspase 3 and PARP cleavage induced by doxorubicin-alkyne and Lqh-8/6-doxorubicin. Total proteins were extracted and detected by Western blot analyses using antibodies against C-Caspase-3 and PARP. (**E**) Up-regulation of Bax and Bim levels in U-87 cells by 10 µM of doxorubicin-alkyne or Lqh-8/6-doxorubicin.

We investigated whether Lqh-8/6-doxorubicin cell toxicity could be attributed to the induction of apoptosis. As shown in Figure 7A, U-87 cells treated with 10 µM of Lqh-8/6-doxorubicin or 10 µM of doxorubicin-alkyne for 24 h displayed similar typical morphologic features of apoptotic cells with chromatin condensation and nuclei fragmentation, as visualized by fluorescence microscopy after DNA staining with Hoechst 33342. These morphologic changes were not observed in the case of Lqh-8/6-azide. Coherent with these observations, it was found that both Lqh-8/6-doxorubicin and doxorubicin-alkyne induce an increase in the percentage of apoptotic cells: 26.8 ± 2.0% for Lqh-8/6-doxorubicin and 27.5 ± 2.0% for doxorubicin-alkyne alone as compared to untreated cells (3.5 ± 1.0%) or cells treated with Lqh-8/6-azide (4.5 ± 1.3%) (Figure 7B). In order to investigate whether doxorubicin-alkyne or Lqh-8/6-doxorubicin-induced apoptosis is mediated by caspases, we first used two caspase inhibitors: a broad-spectrum caspase inhibitor, z-VAD-fmk, and a caspase-3/-7-specific inhibitor, z-DEVD-fmk. The pretreatment of U-87 cells with these caspase inhibitors (100 µM) partially inhibited apoptosis, suggesting that doxorubicin-alkyne and Lqh-8/6-doxorubicin-induced apoptotic cell death is in part dependent on caspase activation (Figure 7C). Furthermore, the proteolytic activation of caspase-3 was examined. Lqh-8/6-azide did not induce caspase-cleavage, whereas 10 µM of doxorubicin-alkyne or Lqh-8/6-doxorubicin caused activation of caspase-3 after 24 h, as indicated by the detection of the cleavage fragments (p20 and p17 fragments) (Figure 7D). The activation of caspase-3 in doxorubicin-alkyne and Lqh-8/6-doxorubicin-treated cells (10 µM) was further confirmed by the cleavage of its known substrate PARP with the appearance of an 89 kDa cleavage fragment (Figure 7D). Moreover, we studied the impact of these compounds on the expression levels of pro-apoptotic proteins (Bax and Bim) in U-87 cells by Western blotting. An important up-regulation of Bax and Bim levels were observed in U-87 cells treated 24 h with 10 µM of doxorubicin-alkyne or Lqh-8/6-doxorubicin (Figure 7E). Taken together, these results demonstrate that Lqh-8/6-doxorubicin induces cell death that is partly mediated by apoptosis with equivalent efficiency than doxorubicin-alkyne alone.

## 4. Discussion

Herein, a @TOMEV2 search for toxins presenting sequence homologies with chlorotoxin—a well-studied peptide for its usefulness in characterizing, diagnosing and treating glioma—revealed the existence of several new scorpion venom peptides that may possess properties akin to chlorotoxin. A total of 14 sequences were identified to possess significant homologies (≥61%). Among these sequences, two had previously been recognized as possessing anti-migration and anti-proliferation properties: AaCtx [51] and BmKCT [42]. These two toxins ranked seventh and thirteenth in terms of sequence homology with chlorotoxin indicating that all the retrieved sequences have the potential to be potent glioma-labeling peptides. With the exception of the N- (before C_1_) and C-termini (after C_8_), the only region that showed amino acid insertions or deletion within the toxin sequences was the one located between C_5_ and C_7_. Most of the observed differences occurred between C_5_ and C_6_, suggesting that this region may not be essential for the activity of the toxins. Three new toxins were chemically synthesized for the first time: Lqh-8/6, lepidopteran and BS-14. These additional syntheses now bring to five the total number of toxins that have been chemically produced on this list of 15 toxins (Table 1). Lqh-8/6 originated from a scorpion that is phylogenetically very close to the scorpion from which chlorotoxin was identified. Lepidopteran and BS-14, together with AaCtx, were last on the list of sequence homologies; they also belong to the same scorpion as BmKCT. All synthesized toxins had the ability to bind to and integrate into GBM cells, validating this search for analogues of chlorotoxin. Quite surprisingly, there are no structure-activity relationship studies published for chlorotoxin or some of its closest analogues. This may be due to the lack of an identified membrane receptor. However, it is obvious that such information would tremendously facilitate the identification of novel toxins possessing chlorotoxin glioma-labeling properties. In the future, chemical engineering work aimed at functionalizing Lqh-8/6 may help with the performance of reverse pharmacology experiments for the identification of the GBM receptor recognizing Lqh-8/6. The identification of the receptor for this toxin could also help to understand the mechanism of GBM invasion on which this toxin is intrinsically active.

Similar to chlorotoxin, there is no doubt that various types of functionalization may be performed on Lqh-8/6 to exploit its specific labeling of GBM cells. As such, many analogues carrying appropriate tags can be envisioned: (i) a fluorescent tag, emitting in the near infrared (such as Cy5.5), for tumor painting during surgery or diagnosis on biopsies; (ii) a chelating agent for contrast agents for MRI; and (iii) a radionuclide such as ^123^iodine or technetium-99 m for tumor treatment. There are also chelating agents with mixed properties that can be grafted onto Lqh-8/6. These agents are versatile in the sense that they accept either a fluorescent lanthanide (terbium, europium) for diagnosis or a short wavelength radionuclide for therapeutic treatment, minimizing the cost of production of Lqh-8/6 analogues. Finally, it is worth mentioning that Lqh-8/6 may also be functionally coupled to nanoparticles for the development of multimodal functions. Examples in the case of chlorotoxin are numerous [52,53,54,55]. Herein, we have provided evidence that functionalization with biotin/streptavidin or doxorubicin through click chemistry does not hinder GMB receptor recognition by Lqh-8/6, thereby indicating that the development of these future analogues should not be problematic. In this respect, the mastering of click chemistry for the grafting of doxorubicin onto Lqh-8/6 serves as a good experimental basis for the grafting of other components of functions onto Lqh-8/6, also by click chemistry, without requiring the synthesis of a new peptide. In addition, the Lqh-8/6_b_-strep-Cy5 or Cy3 can also be useful for the treatment of human biopsies in the future.

As many venom-derived toxins, Lqh-8/6 was found to possess no intrinsic cell toxicity, at least with mammalian cell lines. By itself, it also possesses low anti-tumor properties. Nevertheless, it showed potent inhibition of cell invasion, which is an interesting property to exploit. The most important effect of Lqh-8/6 was its capacity to specifically label GBM cells. Like for chlorotoxin before, it will be of interest to determine whether Lqh-8/6 also labels tumor cells of the same neuroectodermal origin. This cell targeting property was exploited to selectively target an anti-cancer compound to GBM cells. This project required two innovations: (i) the development of a doxorubicin analogue compatible with click chemistry and that preserves, at least partially, its toxic mode of action; (ii) the chemical synthesis of an Lqh-8/6 analogue that is also compatible with click chemistry, that would conjugate efficiently to doxorubicin-alkyne with a high yield, and again, without perturbing the activity of doxorubicin. On all counts, these requirements were successful. The choice of the linker, an additional N-terminal flexible Gly residue along with Aha, was sufficient to preserve doxorubicin action without further optimization. Coupling to doxorubicin conferred to Lqh-8/6 a GBM toxicity that was not observed with the native peptide. In this sense, the conjugation process is a success. The mechanism of cell death induction clearly involves cell apoptosis. Inasmuch as antibody-drug conjugates, these data confirm that toxin conjugates may possess a bright future for the targeted treatment of cancers. This strategy presents the immense advantage of avoiding some of the negative secondary effects that lead to a reduction in the use of doxorubicin in clinics. Conjugation to a targeting peptide should significantly reduce the cardiotoxicity of doxorubicin [56,57]. This example illustrates the benefit of the approach in providing a second potential clinical life to a known anti-tumor agent. In addition, the strategy employed also presents the added benefit of reducing the time of development for FDA approval if clinical assays demonstrate the viability of the approach. While these results were positive, there is room for improvement in the future. The efficacy of Lqh-8/6-doxorubicin on GBM cells in vitro was found to be less than doxorubicin-alkyne alone. This reduction in efficacy is probably linked to a reduced delivery of the doxorubicin moiety with the cytoplasm/nucleus of GBM cells. Part of this reduction may be due to the absence of a cleavable linker between Lqh-8/6 and doxorubicin. Future developments may, therefore, focus on different types of linkers, such as a pH-sensitive hydrazine bond that should more readily liberate doxorubicin in the more acidic environment of the tumor [57,58,59,60]. An ester bond is another alternative that relies on intracellular esterases. Finally, without further modifications, endosome disruption agents may facilitate the escape of a fraction of the toxin conjugate from the endosomes. Their use is, however, limited in vivo. The search for a cleavable linker will hopefully yield a toxin conjugate that has a toxicity profile on GBM cells that is identical to doxorubicin for the next generation of toxin conjugates. Another versatile option for improvement concerns the toxic moiety that is grafted onto Lqh-8/6. While doxorubicin seems a pertinent choice in the case of the GBM cell lines that were tested, other toxic moieties can also be envisioned, further illustrating the versatility of this toxin conjugate approach, which is useful for personalized medicine. Along the toxic moieties of interest, we wish to cite other topoisomerase inhibitors, ^131^I, alkylating agents such as platinum (themselves conjugated) [58], pro-apoptotic peptides, anti-microtubule agents, antimetabolites, and other cytotoxic compounds. The choice of these compounds will be linked to the intrinsic sensitivity of GBM cells to these unconjugated agents, to the benefit of the coupling and the feasibility of the conjugation.

## 5. Conclusions

In conclusion, this study adds credit to the concept that animal venom peptides can be used as warheads for the delivery of anti-tumor agents. A similar usefulness for peptide conjugation was illustrated for cell-penetrating peptides [58,59,60,61,62,63,64]. Natural venom peptides present the unusual benefit of extreme stability in vivo. Peptides from venoms have been shown to possess half lives in plasma that exceed 10 h, which is excessively long for a peptide [65]. The reason for this stability lies behind millions of years of evolution and the existence of a very stable disulfide-bridge organized fold. While we developed the example of a natural peptide that presents sequence homology with chlorotoxin, there is significant hope that other peptides may possess similar virtuous targeting properties on unrelated cancers. Tumor cells generally overexpress a repertoire of ion channels and G protein-coupled receptors, and this is exactly what animal venom toxins target in nature with exquisite selectivity and affinity.

## Figures and Tables

**Table 1:** Alignment of amino acid sequences of chlorotoxin-like peptides. Alignments were performed using @TOME V2. Percentage sequence identity is provided by comparison with chlorotoxin sequence. Disulfide bridge pattern is provided when known.

Toxin	Primary Sequence	Length	Identity	Disulfide Bridge Pattern	Species
Chlorotoxin	MC_1_MPC_2_ FTTDH QMARK C_3_DDC_4_C_5_ GGK-G RGKC_6_Y GPQC_7_L C_8_-R--	36 AA	100%	C_1_-C_4_,C_2_-C_6_,C_3_-C_7_,C_5_-C_8_	*Leiurus quinquestriatus quinquestriatus*
I_3_	MC_1_MPC_2_ FTTDH QTARR C_3_RDC_4_C_5_ GGR-G R-KC_6_F G-QC_7_L C_8_GYD	36 AA	82%	C_1_-C_4_,C_2_-C_6_,C_3_-C_7_,C_5_-C_8_	*Buthus eupeus*
I_4_	MC_1_MPC_2_ FTTDH NMAKK C_3_RDC_4_C_5_ GGN-- -GKC_6_F GPQC_7_L C_8_NR	35 AA	82%	C_1_-C_4_,C_2_-C_6_,C_3_-C_7_,C_5_-C_8_	*Buthus eupeus*
Bs-8	RC_1_KPC_2_ FTTDP QMSKK C_3_ADC_4_C_5_ GGK-G KGKC_6_Y GPQC_7_L C_8_	35 AA	80%	C_1_-C_4_,C_2_-C_6_,C_3_-C_7_,C_5_-C_8_	*Buthus sindicus*
I_5_	MC_1_MPC_2_ FTTDP NMANK C_3_RDC_4_C_5_ GGG-K K--C_6_F GPQC_7_L C_8_NR	35 AA	79%	C_1_-C_4_,C_2_-C_6_,C_3_-C_7_,C_5_-C_8_	*Buthus eupeus*
I_5A_	MC_1_MPC_2_ FTTDP NMAKK C_3_RDC_4_C_5_ GGN-G K--C_6_F GPQC_7_L C_8_NR	35 AA	79%	C_1_-C_4_,C_2_-C_6_,C_3_-C_7_,C_5_-C_8_	*Buthus eupeus*
GaTx1	-C_1_GPC_2_ FTTDH QMEQK C_3_AEC_4_C_5_ GGI-G K--C_6_Y GPQC_7_L C_8_NR	34 AA	79%	C_1_-C_4_,C_2_-C_6_,C_3_-C_7_,C_5_-C_8_	*Leiurus quinquestriatus hebraeus*
BmKCT	-C_1_GPC_2_ FTTDA NMARK C_3_REC_4_C_5_ GGI-G K--C_6_F GPQC_7_L C_8_NRI	35 AA	76%	C_1_-C_4_,C_2_-C_6_,C_3_-C_7_,C_5_-C_8_	*Buthus martensii*
Bm12-b	-C_1_GPC_2_ FTTDA NMARK C_3_REC_4_C_5_ GGN-G K--C_6_F GPQC_7_L C_8_NRE	35 AA	76%	C_1_-C_4_,C_2_-C_6_,C_3_-C_7_,C_5_-C_8_	*Buthus martensii*
Lqh-8/6	RC_1_SPC_2_ FTTDQ QMTKK C_3_YDC_4_C_5_ GGK-G KGKC_6_Y GPQC_7_I C_8_APY	38 AA	72%	C_1_-C_4_,C_2_-C_6_,C_3_-C_7_,C_5_-C_8_	*Leiurus quinquestriatus hebraeus*
I_1_	MC_1_MPC_2_ FTTRP DMAQQ C_3_RAC_4_C_5_ KGR-G K--C_6_F GPQC_7_L C_8_GYD	36 AA	71%	C_1_-C_4_,C_2_-C_6_,C_3_-C_7_,C_5_-C_8_	*Buthus eupeus*
Neurotoxin P2	-C_1_GPC_2_ FTTDP YTESK C_3_ATC_4_C_5_ GGR-G K--C_6_V GPQC_7_L C_8_NRI	35 AA	70%	C_1_-C_4_,C_2_-C_6_,C_3_-C_7_,C_5_-C_8_	*Androctonus mauretanicus mauretanicus*
Lepidopteran	RC_1_GPC_2_ FTTDP QTQAK C_3_SEC_4_C_5_ GRK-G G-VC_6_K GPQC_7_I C_8_GIQ	37 AA	63%	C_1_-C_4_,C_2_-C_6_,C_3_-C_7_,C_5_-C_8_	*Buthus tamulus*
AaCtx	MC_1_IPC_2_ FTTNP NMAAK C_3_NAC_4_C_5_ GSRRG S--C_6_R GPQC_7_I C_8_	34 AA	61%	C_1_-C_4_,C_2_-C_6_,C_3_-C_7_,C_5_-C_8_	*Androctonus australis*
Bs-14	-C_1_GPC_2_ FTKDP ETEKK C_3_ATC_4_C_5_ GGI-G R--C_6_F GPQC_7_L C_8_NRGY	36 AA	61%	C_1_-C_4_,C_2_-C_6_,C_3_-C_7_,C_5_-C_8_	*Buthus sindicus*

## Data Availability

The data presented in this study are available on request from the corresponding author.

## Supplementary Materials

The following supporting information can be downloaded at: https://www.mdpi.com/article/10.3390/biomedicines10102605/s1, Figure S1: superimposition of chlorotoxin analogue backbones; Figure S2: GBM F98 labeling with BS-14b or lepidopteranb; Figure S3: Functional effects of BS-14 and lepidopteran on F98 cells in culture.
