# Supplementary material for: A Conjugate between Lqh-8/6, a Natural Peptide Analogue of Chlorotoxin, and Doxorubicin Efficiently Induces Glioma Cell Death
Source: Biomedicines. 2022 Oct 17;10(10):2605. doi: 10.3390/biomedicines10102605 (PMC9599068; doi:10.3390/biomedicines10102605)
Supplement: Supplementary file 1 [file biomedicines-10-02605-s001.zip › biomedicines-1895764-supplementary.pdf]

A Conjugate between Lqh-8/6, a Natural Peptide Analogue of Chlorotoxin, and Doxorubicin Efficiently Induces Glioma Cell Death

Lucie Dardevet [1,2], Feten Najlaoui [3], Sonia Aroui [4], Mayeul Collot [5], Céline Tisseyre [2,6], Michael W. Pennington [7], Jean-Maurice Mallet [8] and Michel De Waard [1,2,9,*]

[1] L'institut du Thorax, Nantes Université, CNRS, INSERM, 44000 Nantes, France; lucie.dardevet@gmail.com
[2] LabEx "Ion Channels, Science & Therapeutics", 06560 Valbonne, France; celine.tisseyre@cea.fr
[3] Laboratoire des Venins et Biomolécules Thérapeutiques LR11IPT08, Institut Pasteur de Tunis,
Tunis 1002, Tunisia; fatennajlaoui@yahoo.fr
[4] Laboratory of Biochemistry, Molecular Mechanisms and Diseases Research Unit, UR12ES08, Faculty of Medicine, University of Monastir, Monastir 09023, Tunisia; sonia_aroui2002@yahoo.fr
[5] Laboratoire de Bioimagerie et Pathologies, UMR CNRS 7021, 74 Route du Rhin, CS 60024,
67401 Illkirch, France; mayeul.collot@unistra.fr
[6] Université Grenoble Alpes, 621 Avenue Centrale, 38400 Saint-Martin d'Hères, France
[7] Ambiopharm Inc., 1024 Dittman Crt, North Augusta, SC 29842, USA; mike.pennington@ambiopharm.com
[8] Laboratoire des Biomolécules, Ecole Normale Supérieure, PSL University, Sorbonne University, CNRS UMR 7203, 75005 Paris, France; jean-maurice.mallet@ens.psl.eu
[9] Smartox Biotechnology, 6 Rue des Platanes, 38120 Saint-Egrève, France
* Correspondence: michel.dewaard@univ-nantes.fr or dewaard@smartox-biotechnology.com;
Tel.: +33-228-080-076

Supplementary Data

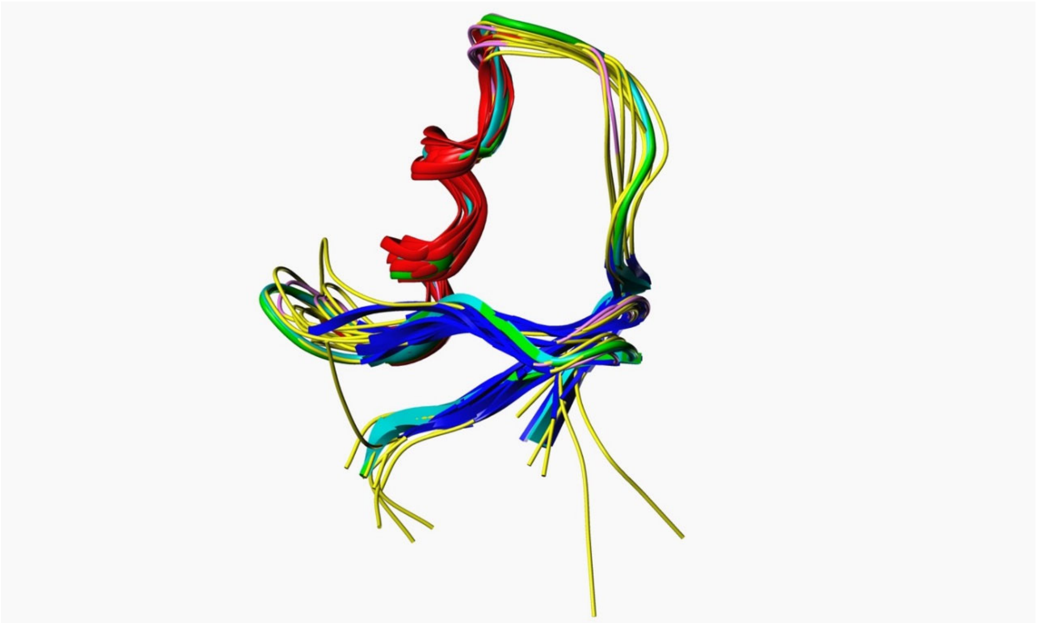

Figure S1. Overlay of the secondary structures of chlorotoxin and chlorotoxin-like peptides. Chlorotoxin is in cyan and Lqh-8/6 in green. The other peptides have the helix alpha in red, and the β-sheet structure is in blue.

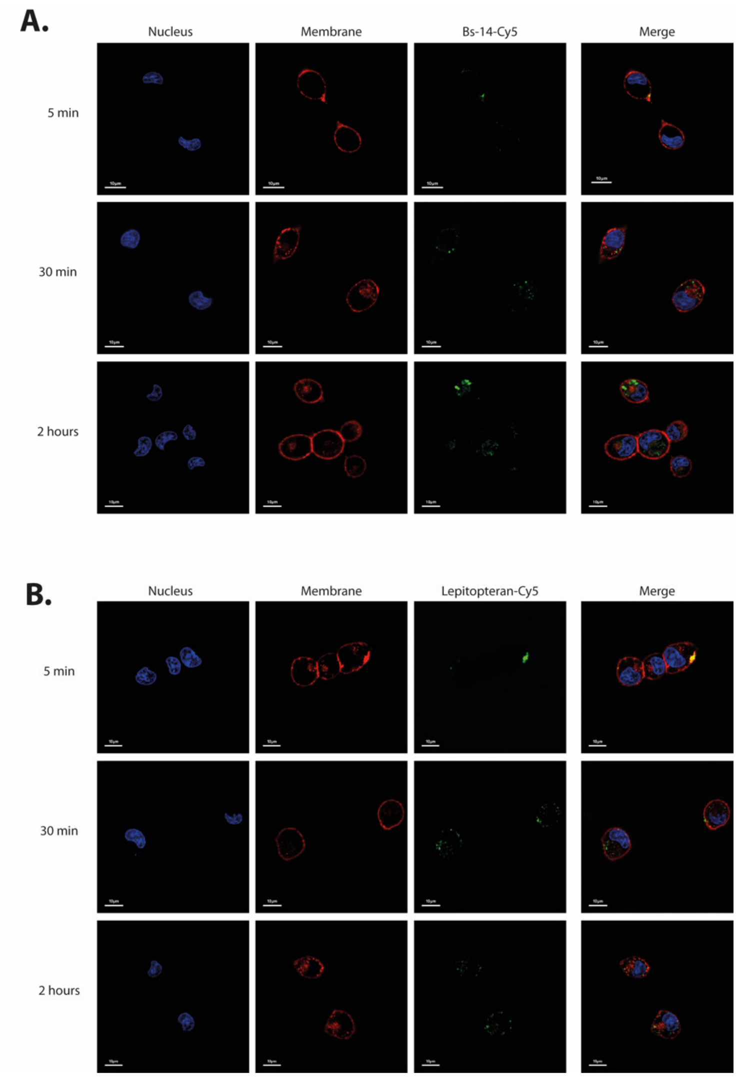

Figure S2. GBM F98 labelling with BS-14$_b$ or lepidopteran$_b$. (**A**) Confocal microscopy images illustrating the cell surface labeling and penetration of BS-14-strep-Cy5 into glioma F98 cells (red color). Incubation times were 5, 30 min and 2 hrs. Images were taken immediately after washout of the extracellular peptide. The plasma membrane is labeled with concanavalin-A-rhodamine (green color); the nucleus is labeled with Hoechst 34580 (blue color). (**B**) Confocal microscopy images illustrating the penetration of lepidopteran$_b$-strep-Cy5 into glioma F98 cells. Same conditions as in (**A**).

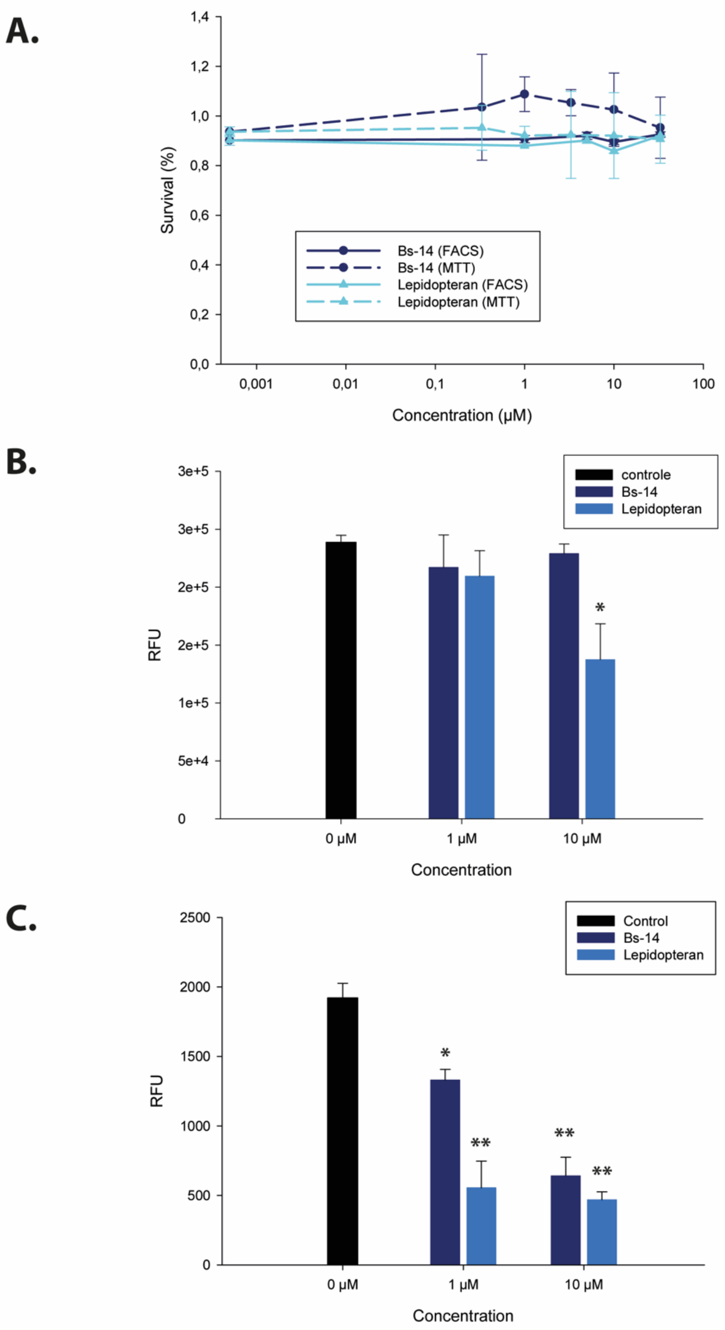

Figure S3. Functional effects of BS-14 and lepidopteran onto F98 cells in culture. (**A**) Lack of cell toxicity of BS-14 and lepidopteran as assessed by MTT assay and FACS. FACS toxicity results are represented by full lines, whereas MTT results are shown with dashed lines. (**B**) Effect of BS-14 and lepidopteran on F98 cell migration. (**C**) Effect of BS-14 and lepidopteran on F98 cell invasion. *, $p \leq 0.07$; **, $p \leq 0.05$.
